# Monotonicity of the ratio of modified Bessel functions of the first kind with applications

**DOI:** 10.1186/s13660-018-1648-4

**Published:** 2018-03-09

**Authors:** Zhen-Hang Yang, Shen-Zhou Zheng

**Affiliations:** 1 Department of Mathematics, Beijing Jiaotong University, Beijing, P.R. China; 2 Present Address: Department of Science and Technology, State Grid Zhejiang Electric Power Company Research Institute, Hangzhou, P.R. China

**Keywords:** 33C10, 39B62, Modified Bessel functions of the first kind, Series, Monotonicity, Inequality

## Abstract

Let $W_{v} ( x ) =xI_{v} ( x ) /I_{v+1} ( x ) $ with $I_{v}$ be the modified Bessel functions of the first kind of order *v*. In this paper, we prove the monotonicity of the function

$$ x\mapsto\frac{ ( W_{v} ( x ) -p ) ^{2}- ( 2v+2-p ) ^{2}}{x^{2}} $$

on $( 0,\infty ) $ for different values of parameter *p* with $v>-2 $. As applications, we deduce some new Simpson–Spector-type inequalities for $W_{v} ( x ) $ and derive a new type of bounds $p+r\sqrt {x^{2}+q^{2}}$ ($r>0$) for $W_{v} ( x ) $. In particular, we show that the upper bound $U_{v-1}^{ ( 2 ) } ( x ) $ for $W_{v} ( x ) $ is the minimum over all upper bounds $\{ U_{p}^{ ( 2 ) } ( x ) :p\leq v-1,v>-2 \} $, where

$$ U_{p}^{ ( 2 ) } ( x ) =p+\sqrt{\frac {2v+2-p}{v+2}x^{2}+ ( 2v+2-p ) ^{2}}, $$

and is not comparable with other sharpest upper bounds. We also find such type of upper bounds for $v-1< p<\min \{ v+1/2,2v+2 \} $ with $v>-2$ and for $2v+2< p< v+1/ ( 2v+5 ) $ with $-2< v<-3/2$.

## Introduction

The modified Bessel functions of the first kind of order *v*, denoted by $I_{v} ( x ) $, are a class of particular solutions of the second-order differential equation [1, p. 77]

1.1 $$ x^{2}\frac{d^{2}y}{dx^{2}}+x\frac{dy}{dx}- \bigl( x^{2}+v^{2} \bigr) y=0, $$

which is represented explicitly by the infinite series

1.2 $$ I_{v} ( x ) =\sum_{n=0}^{\infty} \frac{ ( x/2 ) ^{2n+v}}{n!\Gamma ( v+n+1 ) }=\frac{ ( x/2 ) ^{v}}{\Gamma ( v+1 ) }\sum_{n=0}^{\infty} \frac{ ( x/2 ) ^{2n}}{n! ( v+1 ) _{n}},\quad x\in\mathbb{R}, v\in\mathbb{R}\setminus \{-1,-2,\ldots \}, $$

where

$$ (a)_{n}=a ( a+1 ) \cdots ( a+n-1 ) =\frac{\Gamma(a+n)}{\Gamma(a)}\quad \text{for }n\in\mathbb {N}\text{ and } ( a ) _{0}=1 $$

with $a\neq0,-1,-2,\ldots$ .

It is well known that the ratio $W_{v} ( x ) =xI_{v} ( x ) /I_{v+1} ( x ) $ plays an important role in the finite elasticity [2, 3] and epidemiological models [4, 5]. It was proved in [2, Theorem 2] by Simpson and Spector that $W_{v}$ is strictly increasing and convex on $( 0,\infty ) $ for $v\geq0$, and the inequality

1.3 $$ W_{v} ( x ) ^{2}- ( 2v+1 ) W_{v} ( x ) - \biggl( x^{2}+v+\frac{1}{2} \biggr) >0 $$

holds for $x>0$ and $v\geq0$. For this, such an inequality similar to (1.3) was called Simpson–Spector-type inequality for $W_{v}(x)$ by Yang and Zheng [6, p. 2]. In [7, Proposition 5] Neuman presented a reversed version of (1.3):

1.4 $$ W_{v} ( x ) ^{2}- ( 2v+1 ) W_{v} ( x ) - \biggl( x^{2}+v+\frac{1}{2} \biggr) < v+\frac{3}{2} $$

for $x>0$ and $v>-3/2$. In 2007, Baricz and Neuman [8, Theorem 2.2] extended the range of *v* from $v\geq0$ to $v>-2$ such that $W_{v}$ is strictly increasing on $( 0,\infty ) $, and showed that the inequality

1.5 $$ W_{v} ( x ) ^{2}-2vW_{v} ( x ) -x^{2}>4 ( v+1 ) $$

holds for $x>0$ and $v>-2$. Very recently, Yang and Zheng in [6] got the necessary and sufficient conditions for the Simpson–Spector-type inequality $S_{p,v} ( x ) < u$ or $S_{p,v} ( x ) >l$ to hold for $x>0$ by establishing the monotonicity of $S_{p,v}(x)$ in $x\in ( 0,\infty ) $ with $v>-3/2$, where

$$ S_{p,v} ( x ) =W_{v} ( x ) ^{2}-2pW_{v} ( x ) -x^{2}, $$

which actually answered an open problem recently posed by Hornik and Grün in [9]. Other similar or equivalent inequalities involving the ratio $W_{v} ( x ) $ can be found in [10, Eqs. (11) and (16)], [11], [12, E1. (A.5)], [13], [14, Theorem 1.1], [15, Eqs. (22) and (61)], [9, 16–18] and the references therein.

Motivated by these above-mentioned recent papers, the main aim of this present paper is to prove the monotonicity of the function

1.6 $$ F_{p} ( x ) =\frac{ ( W_{v} ( x ) -p ) ^{2}- ( 2v+2-p ) ^{2}}{x^{2}} $$

on $( 0,\infty ) $ for $v>-2$. Our main result is stated as follows.

### Theorem 1.1

*For* $v>-2$, *let the function* $F_{p}$ *be defined in* $( 0,\infty ) $ *by* (1.6) *and* $c_{v}$ *be defined by*

1.7 $$ c_{v}=\frac{2v^{3}+9v^{2}+9v-4}{2v^{2}+11v+16}. $$

(i) *If* $p\geq v+1/2$ *for* $v\geq-3/2$ *or* $p\geq c_{v}$ *for* $-2< v<-3/2$, *then the function* $F_{p}$ *is increasing from* $( 0,\infty ) $ *onto* $( ( 2v+2-p ) / ( v+2 ) ,1 ) $.
(ii) *If* $p\leq v-1$, *then* $F_{p}$ *is decreasing from* $( 0,\infty ) $ *onto* $( 1, ( 2v+2-p ) / ( v+2 ) ) $.
(iii) *If* $v-1< p< v+1/2$, *then there exists an* $x_{0}>0$ *such that* $F_{p}$ *is increasing on* $( 0,x_{0} ) $, *and decreasing on* $( x_{0},\infty ) $. *Consequently*, *it holds that for* $x>0$,
  1.8 $$ \min \biggl\{ \frac{2v+2-p}{v+2},1 \biggr\} < F_{p} ( x ) < \lambda_{p}, $$
  *where* $\lambda_{p}=F_{p} ( x_{0} ) $, *and* $x_{0}$ *is a unique solution of the equation* $F_{p}^{\prime} ( x ) =0$ *on* $( 0,\infty ) $.
(iv) *If* $v+1/2\leq p< c_{v}$ *for* $-2< v<-3/2$, *then we have*
  1.9 $$ \frac{2v+2-p}{v+2}< F_{p} ( x ) < \theta_{p} $$
  *for* $x>0$, *where* $\theta_{p}=\sup_{x>0}F_{p} ( x ) $. *The lower and upper bounds for* $F_{p} ( x ) $ *are sharp*.

The rest of this paper is organized as follows. In Sect. 2, some lemmas are listed. The proof of Theorem 1.1 is presented in Sect. 3. In Sect. 4, as applications of Theorem 1.1, some Simpson–Spector-type inequalities for $W_{v} ( x ) $ are established in Sect. 4.1; in Sect. 4.2, a new type of bounds $p+r\sqrt{x^{2}+q^{2}}$ ($r>0$) for $W_{v} ( x ) $ for $p<2v+2$ with $v>-2$ is established, and a new Amos-type upper bound $p+\sqrt{x^{2}+q^{2}}$ for $-2< v<-3/2$ is presented; some computable bounds for $W_{v} ( x ) $ for $v-1< p<\min \{ v+1/2,2v+2 \} $ with $v>-2$ and for $2v+2< p< v+1/ ( 2v+5 ) $ with $-2< v<-3/2$ are found in Sect. 4.3.

## Lemmas

To prove Theorem 1.1, we need some lemmas. The following lemma which comes from [19, (3.5)] (see also [20]) is useful.

### Lemma 2.1

*Let* $I_{v}$ *be the modified Bessel function of the first kind of order* *v*, *which is showed by* (1.2). *Then we have*

2.1 $$ I_{u} ( x ) I_{v} ( x ) =\frac{1}{\Gamma ( u+1 ) \Gamma ( v+1 ) }\sum _{n=0}^{\infty}\frac{ ( u+v+n+1 ) _{n}}{n! ( u+1 ) _{n} ( v+1 ) _{n}} \biggl( \frac {x}{2} \biggr) ^{2n+u+v}. $$

*In particular*, *we have*

2.2 $$ I_{v} ( x ) ^{2}=\frac{1}{\Gamma ( v+1 ) ^{2}}\sum _{n=0}^{\infty}\frac{ ( 2v+n+1 ) _{n}}{n! ( v+1 ) _{n}^{2}} \biggl( \frac{x}{2} \biggr) ^{2n+2v}. $$

### Lemma 2.2

([21])

*Let* $A ( x ) =\sum_{k=0}^{\infty}a_{k}x^{k}$ *and* $B ( x ) =\sum_{k=0}^{\infty }b_{k}x^{k}$ *be two real power series converging on* $( -r,r ) $ *for some* $r>0$ *with* $b_{k}>0$ *for all* *k*. *If the sequence* $\{ a_{k}/b_{k}\}$ *is increasing* (*or decreasing*) *for all* *k*, *then the function* $x\mapsto A ( x ) /B ( x ) $ *is also increasing* (*or decreasing*) *on* $( 0,r ) $.

Lemma 2.2 is a powerful tool to deal with the monotonicity of the ratio between two power series. An improvement of Lemma 2.2 has been presented in [22, Theorem 2.1]. A similar monotonicity rule for the ratio of two Laplace transforms was established in [23, Lemma 4] (see also [24]).

### Lemma 2.3

*Let* $A ( x ) =\sum_{k=0}^{\infty }a_{k}x^{k}$ *and* $B ( x ) =\sum_{k=0}^{\infty}b_{k}x^{k}$ *be two real power series converging on* $\mathbb{R}$ *with* $b_{k}>0$ *for all* *k*. *If*, *for certain* $m\in \mathbb{N}$, *the non*-*constant sequence* $\{a_{k}/b_{k}\}$ *is increasing* (*or decreasing*) *for* $0\leq k\leq m$ *and decreasing* (*or increasing*) *for* $k\geq m$, *then there is a unique* $x_{0}\in ( 0,\infty ) $ *such that the function* $A/B$ *is increasing* (*or decreasing*) *on* $( 0,x_{0} ) $ *and decreasing* (*or increasing*) *on* $( x_{0},\infty ) $.

Lemma 2.3 first appeared in [25, Lemma 6.4] without giving the details of the proof. Two strict proofs were given in [22] and [26]. Another useful tool associated with Lemma 2.3 is the sign rule of a class of special series or polynomials, see, for example, [25, Lemma 6.3], [27, Lemma 7], [28]).

### Lemma 2.4

([29, Problems 85, 94])

*If two given sequences* $\{u_{n}\}_{n\geq0}$ *and* $\{v_{n}\}_{n\geq0}$ *satisfy the following conditions*:

$$ v_{n}>0, \qquad\sum_{n=0}^{\infty}v_{n}t^{n} \quad\textit{converges for all values of }t,\quad \textit{and}\quad \lim_{n\rightarrow\infty} \frac{u_{n}}{v_{n}}=s; $$

*then* $\sum_{n=0}^{\infty}u_{n}t^{n}$ *must be convergent for all values of* *t* *too*, *and*

$$ \lim_{t\rightarrow\infty}\frac{\sum_{n=0}^{\infty }u_{n}t^{n}}{\sum_{n=0}^{\infty}v_{n}t^{n}}=s. $$

## Proof of Theorem 1.1

Now we are in a position to prove Theorem 1.1.

### Proof

Let us write $F_{p} ( x ) $ as follows:

$$ F_{p} ( x ) =\frac{x^{2}I_{v} ( x ) ^{2}-2pxI_{v} ( x ) I_{v+1} ( x ) +4 ( v+1 ) ( p-v-1 ) I_{v+1} ( x ) ^{2}}{x^{2}I_{v+1} ( x ) ^{2}}:=\frac{f_{1} ( x ) }{f_{2} ( x ) }, $$

where

$$\begin{aligned}& f_{1} ( x ) =x^{2}I_{v} ( x ) ^{2}-2pxI_{v} ( x ) I_{v+1} ( x ) +4 ( v+1 ) ( p-v-1 ) I_{v+1} ( x ) ^{2}, \\& f_{2} ( x ) =x^{2}I_{v+1} ( x ) ^{2}. \end{aligned}$$

Using formulas (2.1) and (2.2), we have

$$\begin{aligned} f_{1} ( x ) =&\frac{1}{\Gamma ( v+1 ) ^{2}}\sum_{n=0}^{\infty} \biggl[ 4\frac{ ( n+2v+1 ) _{n}}{n! ( v+1 ) _{n}^{2}}-4p\frac{ ( n+2v+2 ) _{n}}{n! ( v+1 ) _{n+1} ( v+1 ) _{n}} \\ &{} +4 ( v+1 ) ( p-v-1 ) \frac{ ( n+2v+3 ) _{n}}{n! ( v+1 ) _{n+1}^{2}} \biggr] \biggl( \frac {x}{2} \biggr) ^{2n+2v+2} \\ :=&\frac{4 ( x^{2}/4 ) ^{v+1}}{\Gamma ( v+1 ) ^{2}}\sum_{n=1}^{\infty}a_{n} \biggl( \frac{x^{2}}{4} \biggr) ^{n}, \end{aligned}$$

where

$$ a_{n}=n\frac{n^{2}+ ( 5v-2p+4 ) n+ ( v+1 ) ( 4v+1 ) -p ( 2v+1 ) }{ ( 2n+2v+1 ) ( n+v+1 ) ( n+2v+2 ) }\frac{ ( n+2v+2 ) _{n}}{n! ( v+1 ) _{n}^{2}}; $$

and

$$\begin{aligned} f_{2} ( x ) =&x^{2}I_{v+1} ( x ) ^{2}= \frac {1}{\Gamma ( v+2 ) ^{2}}x^{2}\sum_{n=0}^{\infty} \frac{ ( n+2v+3 ) _{n}}{n! ( v+2 ) _{n}^{2}} \biggl( \frac{x}{2} \biggr) ^{2n+2v+2} \\ :=&\frac{4 ( x^{2}/4 ) ^{v+1}}{\Gamma ( v+1 ) ^{2}}\sum_{n=1}^{\infty}b_{n} \biggl( \frac{x^{2}}{4} \biggr) ^{n}, \end{aligned}$$

where

$$ b_{n}=\frac{n}{2n+2v+1}\frac{ ( n+2v+2 ) _{n}}{n! ( v+1 ) _{n}^{2}}. $$

Therefore, $F_{p} ( x ) $ can be written in the form of

$$ F_{p} ( x ) =\frac{\frac{4(x^{2}/4)^{v+1}}{\Gamma ( v+1 ) ^{2}}\sum_{n=1}^{\infty}a_{n} ( \frac {x^{2}}{4} ) ^{n}}{\frac{4(x^{2}/4)^{v+1}}{\Gamma ( v+1 ) ^{2}}\sum_{n=1}^{\infty }b_{n} ( \frac{x^{2}}{4} ) ^{n}}=\frac{\sum_{n=0}^{\infty }a_{n+1} ( x^{2}/4 ) ^{n}}{\sum_{n=0}^{\infty}b_{n+1} ( x^{2}/4 ) ^{n}}. $$

A direct computation yields

3.1 $$\begin{aligned} \frac{a_{n}}{b_{n}} =& n\frac{n^{2}+ ( 5v-2p+4 ) n+ ( v+1 ) ( 4v+1 ) -p ( 2v+1 ) }{ ( 2n+2v+1 ) ( n+v+1 ) ( n+2v+2 ) } \big/ \frac {n}{2n+2v+1} \\ =&\frac{n^{2}+ ( 5v-2p+4 ) n+ ( v+1 ) ( 4v+1 ) -p ( 2v+1 ) }{ ( n+2v+2 ) ( n+v+1 ) }; \end{aligned}$$

and from Lemma 2.4 we get

3.2 $$ F_{p} ( 0 ) =\frac{a_{1}}{b_{1}}=\frac{2v+2-p}{v+2}\quad \text{and} \quad F_{p} ( \infty ) =\lim_{n\rightarrow \infty} \frac{a_{n}}{b_{n}}=1. $$

Therefore, to show the monotonicity of the ratio $f_{1}/f_{2}$, it suffices to observe the monotonicity of the sequence $\{a_{n}/b_{n}\}_{n\geq1}$. Since $b_{n}>0$ for $n\geq1$ and $v>-2$, we have

3.3 $$ \frac{a_{n+1}}{b_{n+1}}-\frac{a_{n}}{b_{n}}=\frac{2n^{2}+4 ( v+1 ) n+v ( 2v+3 ) }{ ( n+2v+2 ) ( n+2v+3 ) ( n+v+1 ) ( n+v+2 ) } \bigl[ p-g_{n} ( v ) \bigr] , $$

where

3.4 $$\begin{aligned}& g_{n} ( v ) =\frac{ ( 2v+1 ) n^{2}+ ( 4v^{2}+4v-1 ) n+ ( 2v+3 ) ( v-2 ) ( v+1 ) }{2n^{2}+4 ( v+1 ) n+v ( 2v+3 ) }, \\& \begin{aligned}[b] g_{n+1} ( v ) -g_{n} ( v ) &= \frac{2 ( 2v+3 ) ( n+v+2 ) ( n+2v+3 ) }{ [ 2n^{2}+4 ( v+1 ) n+v ( 2v+3 ) ] [ 2n^{2}+4 ( v+2 ) n+ ( v+2 ) ( 2v+3 ) ] } \\ & \textstyle\begin{cases} \geq0 & \text{if }v\geq-\frac{3}{2}\text{ and }n\geq1, \\ =\frac{4 ( v+3 ) }{2v^{2}+11v+16}>0 & \text{if }-2< v< -\frac {3}{2}\text{ and }n=1, \\ < 0 & \text{if }-2< v< -\frac{3}{2}\text{ and }n\geq2. \end{cases}\displaystyle \end{aligned} \end{aligned}$$

This shows that the sequence $\{g_{n} ( v ) \}_{n\geq1}$ is increasing if $v\geq-3/2$, and increasing for $n=1,2$ then decreasing for $n\geq2$ if $-2< v<-3/2$. Consequently, we deduce that for $n\geq1$,

3.5 $$ v-1=g_{1} ( v ) \leq g_{n} ( v ) < g_{\infty } ( v ) =v+\frac{1}{2} $$

if $v\geq-3/2$ and

3.6 $$ v-1=g_{1} ( v ) < v+\frac{1}{2}=g_{\infty} ( v ) < g_{n} ( v ) < g_{2} ( v ) =c_{v} $$

if $-2< v<-3/2$, where $c_{v}$ is given by (1.7).

Now we discuss the monotonicity of $F_{p}$ by dividing it into two cases.

*Case 1.* $v\geq-3/2$.

*Subcase 1.1.* $p\geq g_{\infty} ( v ) =v+1/2$. From relation (3.3) it is obtained that the sequence $\{ a_{n}/b_{n}\}_{n\geq1}$ is increasing. By Lemma 2.2 it follows that the ratio $f_{1}/f_{2}$ is increasing on $( 0,\infty ) $.

*Subcase 1.2.* $p\leq g_{1} ( v ) =v-1$. It is seen that the sequence $\{a_{n}/b_{n}\}_{n\geq1}$ is decreasing, and from Lemma 2.2 it follows that the ratio $f_{1}/f_{2}$ is decreasing on $( 0,\infty ) $.

*Subcase 1.3.* $v-1< p< v+1/2$. Noting that the sequence $\{ h_{n} ( v ) \}_{n\geq1}=\{p-g_{n} ( v ) \}_{n\geq1}$ is decreasing, and

$$\begin{aligned}& h_{1} ( v ) = p-g_{1} ( v ) =p- ( v-1 ) >0, \\& h_{\infty} ( v ) < p-g_{\infty} ( v ) =p- \biggl( v+ \frac{1}{2} \biggr) < 0, \end{aligned}$$

it is seen that there exists $n_{0}>1$ such that $h_{n} ( v ) >0$ for $1\leq n\leq n_{0}$, and $h_{n} ( v ) <0$ for $n\geq n_{0}$. This implies that $\{a_{n}/b_{n}\}$ is increasing for $1\leq n\leq n_{0}$ and decreasing for $n\geq n_{0}$. By Lemma 2.3 it is derived that there is $x_{0}>0$ such that the ratio $f_{1}/f_{2}$ is increasing on $( 0,x_{0} ) $ and decreasing on $( x_{0},\infty ) $. Consequently, we have

3.7 $$ \min \biggl\{ \frac{2v+2-p}{v+2},1 \biggr\} =\min \biggl\{ \lim _{x\rightarrow0}\frac{f_{1} ( x ) }{f_{2} ( x ) },\lim_{x\rightarrow \infty} \frac{f_{1} ( x ) }{f_{2} ( x ) } \biggr\} \leq \frac{f_{1} ( x ) }{f_{2} ( x ) }\leq\frac {f_{1} ( x_{0} ) }{f_{2} ( x_{0} ) } $$

for $x>0$, that is, inequalities (1.8) hold.

*Case 2.* $-2< v<-3/2$.

*Subcase 2.1.* $p\geq g_{2} ( v ) =c_{v}$. In the same way, we get that the ratio $f_{1}/f_{2}$ is increasing on $( 0,\infty ) $.

*Subcase 2.2.* $p\leq g_{1} ( v ) =v-1$. Similarly, we find that the ratio $f_{1}/f_{2}$ is decreasing on $( 0,\infty ) $.

*Subcase 2.3.* $v-1=g_{1} ( v ) < p< g_{\infty} ( v ) =v+1/2$. We have

$$ \frac{a_{2}}{b_{2}}-\frac{a_{1}}{b_{1}}=\frac{p- ( v-1 ) }{2 ( v+2 ) ( v+3 ) }>0, $$

and notice that $g_{n} ( v ) \geq g_{\infty} ( v ) =v+1/2$ for $n\geq2$. Hence, we get that for $n\geq2$,

$$ \operatorname{sgn} \biggl( \frac{a_{n+1}}{b_{n+1}}-\frac {a_{n}}{b_{n}} \biggr) = \operatorname{sgn} \bigl( p-g_{n} ( v ) \bigr) \leq v+ \frac{1}{2}- \biggl( v+\frac{1}{2} \biggr) =0. $$

This shows that the sequence $\{a_{n}/b_{n}\}$ is increasing for $n=1,2$ and decreasing for $n\geq2$. By Lemma 2.3 it is derived that there is $x_{0}>0$ such that the ratio $f_{1}/f_{2}$ is increasing on $( 0,x_{0} ) $ and decreasing on $( x_{0},\infty ) $. Therefore, inequality (3.7) holds, which implies inequalities (1.8).

*Subcase 2.4.* $v+1/2=g_{\infty} ( v ) \leq p< g_{2} ( v ) =c_{v}$. We easily check that $a_{1}/b_{1}-a_{1}/b_{1}=0$, and for $n\geq2$,

$$\begin{aligned} \frac{a_{n}}{b_{n}}-\frac{a_{1}}{b_{1}} =&\frac{n^{2}+ ( 5v-2p+4 ) n+ ( v+1 ) ( 4v+1 ) -p ( 2v+1 ) }{ ( n+2v+2 ) ( n+v+1 ) }-\frac{2v+2-p}{v+2} \\ =& ( n-1 ) \frac{ ( n+v ) p-nv-v^{2}+v+2}{ ( v+2 ) ( n+v+1 ) ( n+2v+2 ) } \\ \geq& ( n-1 ) \frac{ ( n+v ) ( v+1/2 ) -nv-v^{2}+v+2}{ ( v+2 ) ( n+v+1 ) ( n+2v+2 ) } \\ =&\frac{1}{2} ( n-1 ) \frac{n+3v+4}{ ( v+2 ) ( n+v+1 ) ( n+2v+2 ) }>0. \end{aligned}$$

This yields

$$ F_{p} ( x ) =\frac{\sum_{n=0}^{\infty}a_{n+1} ( x^{2}/4 ) ^{n}}{\sum_{n=0}^{\infty}b_{n+1} ( x^{2}/4 ) ^{n}}>\frac {\sum_{n=0}^{\infty} ( a_{1}b_{n+1}/b_{1} ) ( x^{2}/4 ) ^{n}}{\sum_{n=0}^{\infty}b_{n+1} ( x^{2}/4 ) ^{n}}= \frac {a_{1}}{b_{1}}=\frac{2v+2-p}{v+2} $$

for $x>0$. Since $F_{p} ( 0 ) =a_{1}/b_{1}$, we see that the inequality is sharp.

The continuity of the function $F_{p} ( x ) $ on $( 0,\infty ) $ together with $F_{p} ( 0 ) =a_{1}/b_{1}$ and $F_{p} ( \infty ) =1$ means that $F_{p} ( x ) $ is bounded on $( 0,\infty ) $, so $\sup_{x>0}F_{p} ( x ) $ exists, which completes the proof. □

### Remark 3.1

In Subcase 2.4: $v+1/2< p< c_{v}$ for $-2< v<-3/2$, we see that the sequence $\{h_{n} ( v ) \}_{n\geq2}=\{ p-g_{n} ( v ) \}_{n\geq2}$ is increasing, and

$$\begin{aligned}& h_{2} ( v ) = p-g_{2} ( v ) =p-c_{v}< 0, \\& h_{\infty} ( v ) < p-g_{\infty} ( v ) =p- \biggl( v+ \frac{1}{2} \biggr) >0, \end{aligned}$$

which implies that there exists $n_{1}>2$ such that $h_{n} ( v ) <0$ for $2\leq n\leq n_{1}$ and $h_{n} ( v ) >0$ for $n\geq n_{1}$. This indicates that the sequence $\{a_{n}/b_{n}\}$ is decreasing for $2\leq n\leq n_{1}$ and increasing for $n\geq n_{1}$. Since

$$ \frac{a_{2}}{b_{2}}-\frac{a_{1}}{b_{1}}=\frac{p- ( v-1 ) }{2 ( v+2 ) ( v+3 ) }>0, $$

we find that the sequence $\{a_{n}/b_{n}\}$ is increasing for $n=1,2$, decreasing for $2\leq n\leq n_{1}$, and increasing for $n\geq n_{1}$.

Clearly, we are not able to describe the monotone pattern of $f_{1}/f_{2}$ by directly using Lemmas 2.2 and 2.3. We here guess that there are two $x_{1}$, $x_{2}$ with $x_{2}>x_{1}>0$ such that $f_{1}/f_{2}$ is increasing on $( 0,x_{1} ) \cup ( x_{2},\infty ) $ and decreasing on $( x_{1},x_{2} ) $.

## Some new type of bounds for $W_{v} ( x ) $

### Simpson–Spector-type inequality for $W_{v} ( x ) $

It is clear that

$$ F_{p} ( x ) < ( > ) c\quad \iff\quad \bigl( W_{v} ( x ) -p \bigr) ^{2}- ( 2v+2-p ) ^{2}< ( > ) cx^{2}, $$

where the latter indeed offers some new Simpson–Spector-type inequalities for $W_{v} ( x ) $. In fact, by Theorem 1.1 we immediately get the following.

#### Proposition 4.1

*Let*

$$\begin{aligned}& E_{1} = \biggl\{ p\geq v+\frac{1}{2},v\geq- \frac{3}{2} \biggr\} ,\qquad E_{2}= \biggl\{ p\geq c_{v},-2< v< -\frac{3}{2} \biggr\} , \\& E_{3} = \{ p\leq v-1,v>-2 \} ,\qquad E_{4}= \biggl\{ v-1< p< v+\frac{1}{2},v>-2 \biggr\} , \\& E_{5} = \biggl\{ v+\frac{1}{2}\leq p< c_{v},-2< v< - \frac{3}{2} \biggr\} , \end{aligned}$$

*where* $c_{v}$ *is given in* (1.7). *Then the double inequality*

4.1 $$ \alpha x^{2}< \bigl( W_{v} ( x ) -p \bigr) ^{2}- ( 2v+2-p ) ^{2}< \beta x^{2} $$

*holds for* $x>0$ *and* $v>-2$ *if and only if*

$$\begin{aligned}& \alpha\leq l ( p ) =\min \biggl\{ \frac {2v+2-p}{v+2},1 \biggr\} , \\& \beta\geq u ( p ) = \textstyle\begin{cases} 1 & \textit{if } ( p,v ) \in E_{1}\cup E_{2}, \\ \frac{2v+2-p}{v+2} & \textit{if } ( p,v ) \in E_{3}, \\ \lambda_{p} & \textit{if } ( p,v ) \in E_{4}, \\ \theta_{p} & \textit{if } ( p,v ) \in E_{5}, \end{cases}\displaystyle \end{aligned}$$

*where* $\theta_{p}=\sup_{x>0}F_{p} ( x ) $ *if* $( p,v ) \in E_{5}$, *and*

$$ \lambda_{p}=\frac{ ( W_{v} ( x_{0} ) -p ) ^{2}- ( 2v+2-p ) ^{2}}{x_{0}^{2}}, $$

*and here* $x_{0}$ *is the unique solution of the equation*

4.2 $$ y^{3}- ( p+2v+1 ) y^{2}- \bigl( x^{2}-2pv \bigr) y+px^{2}+4 ( v+1 ) ( p-v-1 ) =0 $$

*on* $( 0,\infty ) $ *with* $y=W_{v} ( x ) $.

#### Proof

(i) By Theorem 1.1 we see that the left-hand side inequality of (4.1) holds for $x>0$ if and only if

$$\begin{aligned} \alpha \leq& \textstyle\begin{cases} \frac{2v+2-p}{v+2} & \text{if } ( p,v ) \in E_{1}\cup E_{2}, \\ 1 & \text{if } ( p,v ) \in E_{3}, \\ \min \{ \frac{2v+2-p}{v+2},1 \} & \text{if } ( p,v ) \in E_{4}, \\ \frac{2v+2-p}{v+2} & \text{if } ( p,v ) \in E_{5}. \end{cases}\displaystyle \\ =& \textstyle\begin{cases} \frac{2v+2-p}{v+2}, & \text{if } ( p,v ) \in E_{1}\cup E_{2}\cup E_{5}\cup ( E_{4}\cap \{ p\geq v,v>-2 \} ) , \\ 1, & \text{if } ( p,v ) \in E_{3}\cup ( E_{4}\cap \{ p\leq v,v>-2 \} ) . \end{cases}\displaystyle \end{aligned}$$

It is easy to check that

$$\begin{aligned} &E_{1}\cup E_{2}\cup E_{5}\cup \bigl( E_{4}\cap \{ p\geq v,v>-2 \} \bigr) = \{ p\geq v,v>-2 \} , \\ &E_{3}\cup \bigl( E_{4}\cap \{ p\leq v,v>-2 \} \bigr) = \{ p\leq v,v>-2 \} , \end{aligned} $$

which indicate that $\alpha\leq l ( p ) $.

(ii) The necessary and sufficient conditions for the right-hand side inequality of (4.1) to hold are obvious.

(iii) As shown in Simpson and Spector [2], $W_{v}$ satisfies the Riccati equation

$$ xW_{v}^{\prime} ( x ) =x^{2}+2 ( v+1 ) W_{v} ( x ) -W_{v} ( x ) ^{2}. $$

Then

$$\begin{aligned} \frac{x^{3}}{2}F_{p}^{\prime} ( x ) =& \bigl( W_{v} ( x ) -p \bigr) xW_{v}^{\prime} ( x ) - \bigl( W_{v} ( x ) -p \bigr) ^{2}+ ( 2v+2-p ) ^{2} \\ =& ( y-p ) \bigl( x^{2}+2 ( v+1 ) y-y^{2} \bigr) - ( y-p ) ^{2}+ ( 2v+2-p ) ^{2} \\ =&-y^{3}+ ( p+2v+1 ) y^{2}+ \bigl( x^{2}-2pv \bigr) y-px^{2}-4 ( v+1 ) ( p-v-1 ) , \end{aligned}$$

where $y=W_{v} ( x ) $. Clearly, if $x_{0}$ is the unique solution of the equation $F_{p}^{\prime} ( x ) =0$ on $( 0,\infty ) $, then so is equation (4.2) on $( 0,\infty ) $.

This completes the proof. □

#### Remark 4.2

Taking $p=v+1/2$ in Proposition 4.1 gives

$$ \frac{2v+3}{2v+4}x^{2}< W_{v} ( x ) ^{2}- ( 2v+1 ) W_{v} ( x ) -2 ( v+1 ) < x^{2}\quad\text{for }x>0\text{ and }v>-\frac{3}{2}, $$

where the left-hand side inequality holds for $x>0$ and $v>-2$, the right-hand side one is inequality (1.4).

Setting $p=v$ in Proposition 4.1 yields

$$ x^{2}< W_{v} ( x ) ^{2}-2vW_{v} ( x ) -4 ( v+1 ) < \lambda_{v}x^{2}\quad\text{for }x>0\text{ and }v>-2, $$

where the left-hand side inequality is inequality (1.5).

In addition, putting $p=c_{v}$ with $-2< v<-3/2$ in Proposition 4.1, where $c_{v}$ is given in (1.7), we obtain a new Simpson–Spector-type inequality, which is stated as a corollary.

#### Corollary 4.3

*Let* $-2< v<-3/2$. *Then the double inequalities*

$$\begin{aligned} &\frac{ ( 2v+3 ) ( v+3 ) ( v+4 ) }{ ( v+2 ) ( 2v^{2}+11v+16 ) }x^{2}\\ &\quad < W_{v} ( x ) ^{2}-2 \frac{2v^{3}+9v^{2}+9v-4}{2v^{2}+11v+16}W_{v} ( x ) -8\frac{ ( 2v+5 ) ( v+1 ) ( v+2 ) }{2v^{2}+11v+16}< x^{2} \end{aligned} $$

*hold for* $x>0$. *The lower and upper bounds are sharp*.

### Sharp bounds for $W_{v} ( x ) $ in the form of $p+r\sqrt{x^{2}+q^{2}}$

A bound in the form of

4.3 $$ A_{p,q} ( x ) =p+\sqrt{x^{2}+q^{2}} $$

for the ratio $W_{v} ( x ) $ is known as Amos-type bound (see [6, 9, 10]). In this subsection, we will give another type of bounds in the form of

4.4 $$ B_{p,q,r} ( x ) =p+r\sqrt{x^{2}+q^{2}} $$

for $W_{v} ( x ) $ by Proposition 4.1. Clearly, $B_{p,q,1} ( x ) =A_{p,q} ( x ) $.

As mentioned in Introduction, Baricz and Neuman [8, Theorem 2.2] (also see [6, Lemma 4.2.]) have shown that $W_{v}$ is strictly increasing from $( 0,\infty ) $ onto $( 2v+2,\infty ) $ for $v>-2$. This implies that $W_{v} ( x ) -p>0$ for $p<2v+2$, and then the double inequality of (4.1) is equivalent to

4.5 $$ p+\sqrt{\alpha x^{2}+ ( 2v+2-p ) ^{2}}< W_{v} ( x ) < p+\sqrt{\beta x^{2}+ ( 2v+2-p ) ^{2}} $$

for $x>0$ and $p<2v+2$ with $v>-2$. Thus from Proposition 4.1 we derive the following statement.

#### Proposition 4.4

*Let* $E_{0}= \{ p<2v+2,v>-2 \} $. (*i*) *The double inequality* (4.5) *holds for* $x>0$ *and* $( p,v ) \in E_{0}$ *if and only if*

$$\begin{aligned}& \alpha\leq l ( p ) =\min \biggl\{ \frac {2v+2-p}{v+2},1 \biggr\} , \\& \beta\geq u^{\ast} ( p ) = \textstyle\begin{cases} 1 & \textit{if }p\geq v+\frac{1}{2},v\geq-\frac{3}{2}, \\ \frac{2v+2-p}{v+2} & \textit{if }p\leq v-1,v>-2, \\ \lambda_{p} & \textit{if }v-1< p< \min \{v+\frac{1}{2},2v+2 \} ,v>-2, \end{cases}\displaystyle \end{aligned}$$

*where* $\lambda_{p}$ *is as in Proposition *4.1.

(*ii*) *Furthermore*, *let*

4.6 $$\begin{aligned}& L_{p} ( x ) =p+\sqrt{\alpha_{\max}x^{2}+ ( 2v+2-p ) ^{2}}, \end{aligned}$$

4.7 $$\begin{aligned}& U_{p} ( x ) =p+\sqrt{\beta_{\min}x^{2}+ ( 2v+2-p ) ^{2}}. \end{aligned}$$

*Then we have*

4.8 $$\begin{aligned}& \max_{p< 2v+2,v>-2}L_{p} ( x ) =v+\sqrt{x^{2}+ ( v+2 ) ^{2}}=L_{v} ( x ) , \end{aligned}$$

4.9 $$\begin{aligned}& \min_{p\geq v+1/2,v\geq-3/2}U_{p} ( x ) =v+\frac {1}{2}+ \sqrt{x^{2}+ \biggl( v+\frac{3}{2} \biggr) ^{2}}:=U_{v+1/2}^{ ( 1 ) } ( x ) , \end{aligned}$$

4.10 $$\begin{aligned}& \min_{p\leq v-1,v>-2}U_{p} ( x ) =v-1+\sqrt{ \frac {v+3}{v+2}x^{2}+ ( v+3 ) ^{2}}:=U_{v-1}^{ ( 2 ) } ( x ) . \end{aligned}$$

*Moreover*, $\min_{p\geq v+1/2,v\geq-3/2}U_{p} ( x ) $ *and* $\min_{p\leq v-1,v\geq-3/2}U_{p} ( x ) $ *are not comparable for* $x\in ( 0,\infty ) $.

#### Proof

(i) By Proposition 4.1, the necessary and sufficient condition such that the left-hand side inequality of (4.5) holds for $x>0$ and $( p,v ) \in E_{0}$ is clear.

While the right-hand side inequality of (4.5) holds for $x>0$ and $( p,v ) \in E_{0}$ if and only if

$$ \beta\geq \textstyle\begin{cases} 1 & \text{if } ( p,v ) \in ( E_{1}\cup E_{2} ) \cap E_{0}, \\ \frac{2v+2-p}{v+2} & \text{if } ( p,v ) \in E_{3}\cap E_{0}, \\ \lambda_{p} & \text{if } ( p,v ) \in E_{4}\cap E_{0}, \\ \theta_{p} & \text{if } ( p,v ) \in E_{5}\cap E_{0}, \end{cases} $$

where $E_{i}$ ($i=1-5$) are given in Proposition 4.1. Simplifying yields $E_{1}\cap E_{0}=E_{1}$, $E_{2}\cap E_{0}=\emptyset$, $E_{3}\cap E_{0}=E_{3}$, $E_{5}\cap E_{0}=\emptyset$, and

$$ E_{4}\cap E_{0}= \biggl\{ v-1< p< \min \biggl\{ v+ \frac{1}{2},2v+2 \biggr\} ,v>-2 \biggr\} , $$

which imply that $\beta\geq u^{\ast} ( p ) $.

(ii) To prove the second assertion of this proposition, we first note that the function

$$ p\mapsto p+\sqrt{x^{2}+ ( 2v+2-p ) ^{2}} $$

is increasing on $\mathbb{R}$, and another function

$$ p\mapsto p+\sqrt{\frac{2v+2-p}{v+2}x^{2}+ ( 2v+2-p ) ^{2}} $$

is decreasing on $(-\infty,2v+2]$.

Now, since

$$ L_{p} ( x ) = \textstyle\begin{cases} p+\sqrt{\frac{2v+2-p}{v+2}x^{2}+ ( 2v+2-p ) ^{2}} & \text{if }v\leq p\leq2v+2, \\ p+\sqrt{x^{2}+ ( 2v+2-p ) ^{2}} & \text{if }p\leq v, \end{cases} $$

the function $p\mapsto L_{p} ( x ) $ is increasing on $(-\infty,v]$ and decreasing on $[ v,2v+2 ] $, which implies $\max_{p\leq 2v+2,v>-2}L_{p} ( x ) =L_{v} ( x ) $.

If $p\geq v+1/2$ with $v\geq-3/2$, then $\beta_{\min}=1$, and then

4.11 $$ U_{p} ( x ) =p+\sqrt{x^{2}+ ( 2v+2-p ) ^{2}}:=U_{p}^{ ( 1 ) } ( x ) , $$

which is increasing in *p* on $[ v+1/2,2v+2 ] $. This gives $\min_{p\geq v+1/2,v\geq-3/2}U_{p} ( x ) =U_{v+1/2}^{ ( 1 ) } ( x ) $.

If $p\leq v-1$ with $v>-2$, then $\beta_{\min}= ( 2v+2-p ) / ( v+2 ) $, and therefore,

4.12 $$ U_{p} ( x ) =p+\sqrt{\frac{2v+2-p}{v+2}x^{2}+ ( 2v+2-p ) ^{2}}:=U_{p}^{ ( 2 ) } ( x ) , $$

which is decreasing in *p* on $(-\infty,2v+2]$. This leads to $\min_{p\leq v-1,v>-2}U_{p} ( x ) =U_{v-1}^{ ( 2 ) } ( x ) $.

Finally, we show that $\min_{p\geq v+1/2,v\geq-3/2}U_{p} ( x ) $ is not comparable with $\min_{p\leq v-1,v\geq-3/2}U_{p} ( x ) $ for $x\in ( 0,\infty ) $. In fact, we have that for $v\geq-3/2$,

$$\begin{aligned}& U_{v+1/2}^{ ( 1 ) } ( x ) -U_{v-1}^{ ( 2 ) } ( x ) = \frac{3}{2}+\sqrt{x^{2}+ \biggl( v+\frac {3}{2} \biggr) ^{2}}-\sqrt{\frac{v+3}{v+2}x^{2}+ ( v+3 ) ^{2}}, \\& \biggl( \frac{3}{2}+\sqrt{x^{2}+ \biggl( v+\frac{3}{2} \biggr) ^{2}} \biggr) ^{2}-\frac{v+3}{v+2}x^{2}- ( v+3 ) ^{2} \\& \quad =3\sqrt{x^{2}+ \biggl( v+\frac{3}{2} \biggr) ^{2}}- \biggl( 3 \biggl( v+\frac{3}{2} \biggr) + \frac{x^{2}}{v+2} \biggr) , \\& \biggl( 3\sqrt{x^{2}+ \biggl( v+\frac{3}{2} \biggr) ^{2}} \biggr) ^{2}- \biggl( 3 \biggl( v+\frac{3}{2} \biggr) +\frac{x^{2}}{v+2} \biggr) ^{2}=-x^{2} \frac{x^{2}-3 ( v+2 ) ( v+3 ) }{ ( v+2 ) ^{2}}. \end{aligned}$$

From this it is seen that $U_{v+1/2}^{ ( 1 ) } ( x ) < U_{v-1}^{ ( 2 ) } ( x ) $ if $x>\sqrt{3 ( v+2 ) ( v+3 ) }$ and $U_{v+1/2}^{ ( 1 ) } ( x ) >U_{v-1}^{ ( 2 ) } ( x ) $ if $0< x<\sqrt{3 ( v+2 ) ( v+3 ) }$.

This completes the proof. □

#### Remark 4.5

Amos [10, Eq. (11)] offered a lower bound $A_{v,v+2} ( x ) < W_{v} ( x ) $ for $x>0$ and $v\geq0$. Hornik and Grün [9, Theorem 6] showed that the Amos-type bound is the sharpest for $x>0$ and $v>-1$. Yang and Zheng [6, Theorem 4.6] extended the range of *v* from $v>-1$ to $v>-3/2$. Proposition 4.4 presents another lower bound $L_{p} ( x ) $ defined in (4.6) for $W_{v} ( x ) $ for $x>0$ with $v>-2$ and shows that $L_{v} ( x ) $ defined by (4.8) is the maximum over all lower bounds $\{ L_{p} ( x ) :p<2v+2,v>-2 \} $. It should be emphasized that our sharpest lower bound $L_{v} ( x ) $ extends the range of $A_{v,v+2} ( x ) $ from $v>-3/2$ to $v>-2$ although $L_{v} ( x ) $ and $A_{v,v+2} ( x ) $ have the same expression.

#### Remark 4.6

Amos [10, Eq. (16)] gave an upper bound $W_{v} ( x ) < A_{v+1/2,v+3/2} ( x ) $ for $x>0$ and $v\geq0 $. Hornik and Grün [9, Theorem 3] proved that this Amos-type upper bound is the best for $x>0$ and $v>-1$, where the range of *v* has been extended from $v>-1$ to $v>-3/2$ in [6, Theorem 4.4] by Yang and Zheng. Since $U_{v+1/2}^{ ( 1 ) } ( x ) =A_{v+1/2,v+3/2} ( x ) $, our Proposition 4.4 demonstrates the same result in [6, Theorem 4.4] by a slightly different approach.

#### Remark 4.7

Proposition 4.4 also gives another upper bounds for $W_{v} ( x ) $ by $U_{p}^{ ( 2 ) } ( x ) $ defined in (4.10) for $x>0$ and $p\leq v-1$ with $v>-2$, that is,

4.13 $$ W_{v} ( x ) < p+\sqrt{\frac{2v+2-p}{v+2}x^{2}+ ( 2v+2-p ) ^{2}}=U_{p}^{ ( 2 ) } ( x ) . $$

Not only the above inequalities are valid, but we explain that $U_{v-1}^{ ( 2 ) } ( x ) $ is the minimum over all upper bounds $\{ U_{p}^{ ( 2 ) } ( x ) :p\leq v-1,v>-2 \} $, and $U_{v-1}^{ ( 2 ) } ( x ) $ and $U_{v+1/2}^{ ( 1 ) } ( x ) $ are not comparable in *x* on $( 0,\infty ) $ for $v\geq-3/2$. This indicates that $U_{v-1}^{ ( 2 ) } ( x ) $ for $v>-2$ is indeed a new sharpest upper bound for $W_{v} ( x ) $. Consequently, Proposition 4.4 in fact offers a new type of bounds $p+r\sqrt{x^{2}+q^{2}}$ ($r>0$) for $W_{v} ( x ) $, which is clearly different from the Amos-type bound $A_{p,q} ( x ) =p+\sqrt {x^{2}+q^{2}}$. Moreover, inequality (4.13) is sharp at $x=0$ in view of

$$ W_{v} ( x ) -U_{p}^{ ( 2 ) } ( x ) \thicksim ( 2v+2 ) -p- \vert 2v+2-p \vert =0 $$

as $x\rightarrow0$.

As a direct consequence of Proposition 4.4, we have the following.

#### Corollary 4.8

*If* $v+1/2\leq p<2v+2$ *with* $v\geq-3/2$, *then the double inequality*

4.14 $$ p+\sqrt{\frac{2v+2-p}{v+2}x^{2}+ ( 2v+2-p ) ^{2}}< W_{v} ( x ) < p+\sqrt{x^{2}+ ( 2v+2-p ) ^{2}} $$

*holds for all* $x>0$. *Inequalities* (4.14) *are reversed if* $p\leq v-1$ *with* $v>-2$.

*In particular*, *taking* $p=v+1/2$, $v+1$, $( 2v+2 ) ^{-}$ *and* $p=v-1$, −∞, *we have*

4.15 $$\begin{aligned}& \begin{aligned}[b] &v+\frac{1}{2}+\sqrt{\frac{v+3/2}{v+2}x^{2}+ \biggl( v+ \frac {3}{2} \biggr) ^{2}}\\ &\quad < W_{v} ( x ) < v+ \frac{1}{2}+\sqrt{x^{2}+ \biggl( v+\frac{3}{2} \biggr) ^{2}}\quad\textit{for }v\geq-\frac{3}{2}, \end{aligned} \end{aligned}$$

4.16 $$\begin{aligned}& v+1+\sqrt{\frac{v+1}{v+2}x^{2}+ ( v+1 ) ^{2}}< W_{v} ( x ) < v+1+\sqrt{x^{2}+ ( v+1 ) ^{2}}\quad\textit{for }v>-1, \end{aligned}$$

4.17 $$\begin{aligned}& 2 ( v+1 ) < W_{v} ( x ) < 2v+2+x\quad\textit{for }v\geq- \frac{3}{2}, \end{aligned}$$

4.18 $$\begin{aligned}& v-1+\sqrt{x^{2}+ ( v+3 ) ^{2}}< W_{v} ( x ) < v-1+ \sqrt{\frac{v+3}{v+2}x^{2}+ ( v+3 ) ^{2}}\quad\textit{for }v>-2, \\& 2 ( v+1 ) < W_{v} ( x ) < 2 ( v+1 ) +\frac{1}{2} \frac{x^{2}}{v+2}\quad\textit{for }v>-2. \end{aligned}$$

#### Remark 4.9

The right-hand side in inequality (4.15) for $v\geq 0$ was proved in [10, Eq. (16)] by Amos, and for $v>-3/2$ it follows from Neuman’s inequality (1.4). The right-hand side one in (4.16) for $v\geq0$ is also due to Amos [10, Eq. (11)], which for $v>-1$ was proved by Yuan and Kalbfleisch [12, Eq. (A.5)], and Laforgia and Natalini [14, Theorem 1.1]. While the left-hand side inequality in (4.16) for $v>-1$ was showed by Segura [15, Eq. (61)]. Inequalities (4.17) were proved by Yang and Zheng in [6, Remark 4.9]. Moreover, the rational bounds given in (4.18) appeared in [4, Appendix] for $v>-1$, so the right-hand side inequality of (4.18) can be viewed as a new one in the sense that the range of *v* is extended from $v>-1$ to $v>-2$.

Now let us return to Proposition 4.1. First, if $( p,v ) \in E_{2}= \{ p\geq c_{v},-2< v<-3/2 \} $, then the right-hand side inequality of (4.1) holds for $x>0$ if and only if $\beta\geq1$, which implies that the double inequality

4.19 $$ p-\sqrt{\beta x^{2}+ ( 2v+2-p ) ^{2}}< W_{v} ( x ) < p+\sqrt{\beta x^{2}+ ( 2v+2-p ) ^{2}} $$

holds for $x>0$. Second, according to the guess presented in Remark 3.1, $\theta_{p}=\sup_{x>0}F_{p} ( x ) $ may equal $F_{p} ( \infty ) =1$ for certain $p\in{}[ v+1/2,c_{v})$ with $-2< v<-3/2$. If so, then the right-hand side inequality of (4.1) holds for $x>0$ if and only if $\beta\geq\theta_{p}=1$, which implies that the double inequality (4.19) also holds for $x>0$ and certain $p\in{}[ v+1/2,c_{v})$ with $-2< v<-3/2$, where $\beta=1$ is the best possible constant. In fact, this claim is valid.

#### Proposition 4.10

*Let* $-2< v<-3/2$. *Then the double inequality* (4.19) *holds for* $x>0$ *and* $p\geq c_{v}^{\ast}=v+1/ ( 2v+5 ) $ *with the best constant* $\beta=1$.

#### Proof

(i) For $p\geq c_{v}$, the desired result is evidently valid by Proposition 4.1.

(ii) For $c_{v}^{\ast}\leq p< c_{v}$, where $c_{v}$ is defined by (1.7), it is easy to check that

$$\begin{aligned}& c_{v}^{\ast}- \biggl( v+\frac{1}{2} \biggr) = - \frac{1}{2}\frac {2v+3}{2v+5}>0, \\& c_{v}^{\ast}-c_{v} = 2\frac{ ( 2v+3 ) ( v+2 ) ( v+3 ) }{ ( 2v+5 ) ( 2v^{2}+11v+16 ) }< 0, \end{aligned}$$

which imply $c_{v}^{\ast}\in{}[ c+1/2,c_{v})$. To prove the desired assertion, it suffices to prove that

4.20 $$ F_{p} ( x ) =\frac{f_{1} ( x ) }{f_{2} ( x ) }=\frac{\sum_{n=0}^{\infty}a_{n+1} ( x^{2}/4 ) ^{n}}{\sum_{n=0}^{\infty}b_{n+1} ( x^{2}/4 ) ^{n}}< 1 $$

for $x>0$ and $c_{v}^{\ast}\leq p< c_{v}$ with $-2< v<-3/2$. Indeed, we have

$$ \frac{a_{1}}{b_{1}}-1=-\frac{p-v}{v+2}< 0, $$

and for $n\geq2$,

$$\begin{aligned} \frac{a_{n}}{b_{n}}-1 =&\frac{-p ( 2n+2v+1 ) +n ( 2v+1 ) + ( v+1 ) ( 2v-1 ) }{ ( n+v+1 ) ( n+2v+2 ) } \\ \leq&\frac{-c_{v}^{\ast} ( 2n+2v+1 ) +n ( 2v+1 ) + ( v+1 ) ( 2v-1 ) }{ ( n+v+1 ) ( n+2v+2 ) } \\ =&\frac{ ( n-2 ) ( 2v+3 ) }{ ( n+2v+2 ) ( 2v+5 ) ( n+v+1 ) }\leq0, \end{aligned}$$

which yield

$$ F_{p} ( x ) =\frac{\sum_{n=0}^{\infty}a_{n+1} ( x^{2}/4 ) ^{n}}{\sum_{n=0}^{\infty}b_{n+1} ( x^{2}/4 ) ^{n}}< \frac {\sum_{n=0}^{\infty}b_{n+1} ( x^{2}/4 ) ^{n}}{\sum_{n=0}^{\infty }b_{n+1} ( x^{2}/4 ) ^{n}}=1. $$

In view of $F_{p} ( \infty ) =1$, the upper bound given in (4.20) is sharp, and by Proposition 4.1 the desired assertion follows. Thus we complete the proof. □

#### Remark 4.11

It is easy to check that the lower bound for $W_{v} ( x ) $ given in (4.19) is weaker than $2v+2$, but the upper bound for $\beta=1$ is clearly a new Amos-type bound for $p\geq c_{v}^{\ast}$ with $-2< v<-3/2$ since it is not comparable with the sharpest upper bound $U_{v-1}^{ ( 2 ) } ( x ) $ for $p\geq c_{v}^{\ast}$ with $-2< v<-3/2$, while another one $U_{v+1/2}^{ ( 1 ) } ( x ) $ is restricted in $v\geq-3/2$.

### Some computable bounds for $W_{v} ( x ) $

From Proposition 4.4 we see that the minimum $\beta_{\min }=\lambda _{p}$ for $v-1< p<\min \{ v+1/2,2v+2 \} $ with $v>-2$ such that the inequality

$$ W_{v} ( x ) < p+\sqrt{\lambda_{p}x^{2}+ ( 2v+2-p ) ^{2}}=U_{p}^{ ( 3 ) } ( x ) $$

holds for $x>0$. Since $\lambda_{p}=f_{1} ( x_{0} ) /f_{2} ( x_{0} ) $, where $x_{0}$ is the unique solution of equation (4.2) on $( 0,\infty ) $, the number $\lambda_{p}$ is usually not computable, and so is $U_{p}^{ ( 3 ) } ( x ) $. Therefore, it is interesting and useful to find some upper bounds for $\lambda_{p}$ by elementary functions.

In this subsection, we will find some upper bounds for $\lambda_{p}$ in terms of elementary functions to obtain some computable upper bounds for $W_{v} ( x ) $ by using relation (3.7), that is,

$$ \lambda_{p}=\frac{f_{1} ( x_{0} ) }{f_{2} ( x_{0} ) }>\min \biggl\{ \frac{2v+2-p}{v+2},1 \biggr\} , $$

and an analogous technique used in the proof of Subcase 2.4 of Theorem 1.1.

#### Proposition 4.12

*Let* $v-1< p<\min \{ v+1/2,2v+2 \} $ *with* $v>-2$. *Then the inequality*

$$ W_{v} ( x ) < p+\sqrt{\lambda_{p}^{\ast}x^{2}+ ( 2v+2-p ) ^{2}} $$

*holds for* $x>0$, *where*

4.21 $$ \lambda_{p}^{\ast}=\min \biggl\{ \frac{4v+5-2p}{2 ( v+2 ) }, \frac{v+3}{v+2} \biggr\} . $$

#### Proof

It suffices to prove $a_{n}/b_{n}\leq\lambda_{p}^{\ast}$. We first prove that

$$ \frac{a_{n}}{b_{n}}\leq\frac{4v+5-2p}{2 ( v+2 ) }=1+\frac {v+1/2-p}{v+2}= \frac{2v+2-p}{v+2}+\frac{1}{2}\frac{1}{v+2} $$

holds for all $n\geq1$ by dividing the proof into two cases.

*Case 1.* $\min \{ v+1/2,2v+2 \} =v+1/2$, namely $v\geq -3/2$. For this, we write $a_{n}/b_{n}$ as

$$ \frac{a_{n}}{b_{n}}=\frac{2n+2v+1}{ ( n+2v+2 ) ( n+v+1 ) } \biggl( v+\frac{1}{2}-p \biggr) + \frac{n^{2}+ ( 3v+3 ) n+2v^{2}+3v+1/2}{ ( n+2v+2 ) ( n+v+1 ) }. $$

Then we have

$$\begin{aligned} \frac{a_{n}}{b_{n}}-\frac{4v+5-2p}{2 ( v+2 ) } =&\frac {2n+2v+1}{ ( n+2v+2 ) ( n+v+1 ) } \biggl( v+ \frac {1}{2}-p \biggr) -\frac{v+1/2-p}{v+2} \\ &{}+\frac{n^{2}+ ( 3v+3 ) n+2v^{2}+3v+1/2}{ ( n+2v+2 ) ( n+v+1 ) }-1 \\ =&-\frac{ ( n-1 ) ( n+v ) ( v+1/2-p ) }{ ( v+2 ) ( n+v+1 ) ( n+2v+2 ) }-\frac {v+3/2}{ ( n+2v+2 ) ( n+v+1 ) }< 0 \end{aligned}$$

for $n\geq1$.

*Case 2.* $\min \{ v+1/2,2v+2 \} =2v+2$, namely $-2< v<-3/2$. Similarly, we write $a_{n}/b_{n}$ as

$$ \frac{a_{n}}{b_{n}}=\frac{2n+2v+1}{ ( n+2v+2 ) ( n+v+1 ) } ( 2v+2-p ) +\frac{n-1}{n+2v+2}. $$

Then we get

$$\begin{aligned} \frac{a_{n}}{b_{n}}-\frac{4v+5-2p}{2 ( v+2 ) } &=\frac {2n+2v+1}{ ( n+2v+2 ) ( n+v+1 ) } ( 2v+2-p ) - \frac{2v+2-p}{v+2} \\ &\quad {}+\frac{n-1}{n+2v+2}-\frac{1}{2}\frac{1}{v+2} \\ &=-\frac{ ( n-1 ) ( n+v ) ( 2v+2-p ) }{ ( v+2 ) ( n+v+1 ) ( n+2v+2 ) }+\frac {1}{2}\frac{ ( n-2 ) ( 2v+3 ) }{ ( v+2 ) ( n+2v+2 ) }< 0 \end{aligned} $$

for $n\geq1$.

Second, to prove that for all $n\geq1$,

$$ \frac{a_{n}}{b_{n}}\leq\frac{v+3}{v+2}, $$

we write $a_{n}/b_{n}$ in the form of

$$ \frac{a_{n}}{b_{n}}=\frac{2n+2v+1}{ ( n+2v+2 ) ( n+v+1 ) } ( v-1-p ) +\frac{n^{2}+3 ( v+2 ) n+2v^{2}+6v+2}{ ( n+2v+2 ) ( n+v+1 ) }. $$

Then, for $n\geq1$, we have

$$\begin{aligned} \frac{a_{n}}{b_{n}}-\frac{v+3}{v+2} =&\frac{ ( v-1-p ) ( 2n+2v+1 ) }{ ( n+2v+2 ) ( n+v+1 ) }+ \frac {n^{2}+3 ( v+2 ) n+2v^{2}+6v+2}{ ( n+2v+2 ) ( n+v+1 ) }-\frac{v+3}{v+2} \\ =&\frac{ ( v-1-p ) ( 2n+2v+1 ) }{ ( n+2v+2 ) ( n+v+1 ) }-\frac{ ( n-1 ) ( n-2 ) }{ ( v+2 ) ( n+v+1 ) ( n+2v+2 ) }< 0. \end{aligned}$$

Finally, it is obtained that

$$ \lambda_{p}=\frac{f_{1} ( x_{0} ) }{f_{2} ( x_{0} ) }=\frac{\sum_{n=0}^{\infty}a_{n} ( x_{0}^{2}/4 ) ^{n}}{\sum_{n=0}^{\infty}b_{n} ( x_{0}^{2}/4 ) ^{n}}< \frac{\sum_{n=0}^{\infty}\lambda _{p}^{\ast}b_{n} ( x_{0}^{2}/4 ) ^{n}}{\sum_{n=0}^{\infty }b_{n} ( x_{0}^{2}/4 ) ^{n}}=\lambda_{p}^{\ast }, $$

which completes the proof. □

Now by Proposition 4.12 we have the following.

#### Corollary 4.13

*Let* $v-1< p<\min \{ v+1/2,2v+2 \} $ *with* $v>-2$.

(i) *For* $v-1< p\leq v-1/2$, *the inequality*
  4.22 $$ W_{v} ( x ) < p+\sqrt{\frac{v+3}{v+2}x^{2}+ ( 2v+2-p ) ^{2}}=U_{p}^{\ast\ast} ( x ) $$
  *holds for* $x>0$.
(ii) *For* $v-1/2< p<\min \{ v+1/2,2v+2 \} $, *the inequality*
  4.23 $$ W_{v} ( x ) < p+\sqrt{\frac{4v+5-2p}{2 ( v+2 ) }x^{2}+ ( 2v+2-p ) ^{2}}=U_{p}^{\ast} ( x ) $$
  *holds for* $x>0$. *In particular*, *taking* $p=v$ *and letting* $p\rightarrow v+1/2 $ *with* $v\geq-3/2$ *and* $p\rightarrow2v+2$ *with* $-2< v<-3/2$, *the following inequalities hold for* $x>0$:
  4.24 $$\begin{aligned}& W_{v} ( x ) < v+\sqrt{\frac{2v+5}{2 ( v+2 ) }x^{2}+ ( v+2 ) ^{2}}=U_{v}^{\ast} ( x )\quad \textit{for }v>-2, \end{aligned}$$
  4.25 $$\begin{aligned}& W_{v} ( x ) < v+\frac{1}{2}+\sqrt{x^{2}+ \biggl( v+ \frac {3}{2} \biggr) ^{2}}=U_{v+1/2}^{\ast} ( x ) \quad \textit{for }v\geq-\frac{3}{2}, \end{aligned}$$
  4.26 $$\begin{aligned}& W_{v} ( x ) < 2v+2+\frac{x}{\sqrt{2v+4}}=U_{2v+2}^{\ast } (x )\quad \textit{for }-2< v< -\frac{3}{2}. \end{aligned}$$

#### Remark 4.14

It is easy to check that the function $p\mapsto U_{p}^{\ast \ast} ( x ) $ defined in (4.22) is increasing on $\mathbb{R}$, which yields

$$ U_{p}^{\ast\ast} ( x ) >U_{v-1}^{\ast\ast} ( x ) =v-1+\sqrt{\frac{v+3}{v+2}x^{2}+ ( v+3 ) ^{2}}=U_{v-1}^{ ( 2 ) } ( x ) $$

for $v-1< p\leq v-1/2$ with $v>-2$. This shows that the upper bound $U_{p}^{\ast\ast} ( x ) $ for $W_{v} ( x ) $ is weaker than $U_{v-1}^{ ( 2 ) } ( x ) $ as the sharpest one given in Proposition 4.4. Inequality (4.26) seems to be a new one.

#### Remark 4.15

Clearly, $U_{v+1/2}^{\ast} ( x ) =U_{v+1/2}^{ ( 1 ) } ( x ) $ for $v\geq-3/2$. In general, the upper bound $U_{p}^{\ast} ( x ) $ for $v-1/2< p<\min \{ v+1/2,2v+2 \} $ with $v>-2$ given in (4.23) is not comparable with other two sharpest upper bounds $U_{v+1/2}^{ ( 1 ) }$ for $v\geq-3/2$ and $U_{v-1}^{ ( 2 ) } ( x ) $ for $v>-2$ given in Proposition 4.4. For example, for $v\geq-3/2$, $U_{v}^{\ast } ( x ) < U_{v+1/2}^{ ( 1 ) } ( x ) $ if $0< x<\sqrt {2 ( v+2 ) }$ and $U_{v}^{\ast} ( x ) >U_{v+1/2}^{ ( 1 ) } ( x ) $ if $x>\sqrt{2 ( v+2 ) }$, since

$$\begin{aligned}& \begin{aligned} U_{v}^{\ast} ( x ) -U_{v+1/2} ( x ) &=v+\sqrt {\frac{2v+5}{2 ( v+2 ) }x^{2}+ ( v+2 ) ^{2}}- \biggl( v+\frac{1}{2}+\sqrt{x^{2}+ \biggl( v+ \frac{3}{2} \biggr) ^{2}} \biggr) \\ &=\sqrt{\frac{2v+5}{2 ( v+2 ) }x^{2}+ ( v+2 ) ^{2}}- \biggl( \frac{1}{2}+\sqrt{x^{2}+ \biggl( v+\frac{3}{2} \biggr) ^{2}} \biggr) , \end{aligned} \\& \begin{aligned} &\frac{2v+5}{2 ( v+2 ) }x^{2}+ ( v+2 ) ^{2}- \biggl( \frac{1}{2}+\sqrt{x^{2}+ \biggl( v+\frac{3}{2} \biggr) ^{2}} \biggr) ^{2}\\ &\quad =\frac{ ( v+2 ) ( 2v+3 ) +x^{2}}{2 ( v+2 ) }-\sqrt{x^{2}+ \biggl( v+\frac{3}{2} \biggr) ^{2}}, \end{aligned} \\& \biggl( \frac{ ( v+2 ) ( 2v+3 ) +x^{2}}{2 ( v+2 ) } \biggr) ^{2}- \biggl( x^{2}+ \biggl( v+\frac{3}{2} \biggr) ^{2} \biggr) =\frac{1}{4}x^{2} \frac{x^{2}-2 ( v+2 ) }{ ( v+2 ) ^{2}}. \end{aligned}$$

Similarly, for $v>-2$, $U_{v}^{\ast} ( x ) < U_{v-1}^{ ( 2 ) } ( x ) $ if $x>\sqrt{8 ( v+2 ) ( v+3 ) }$ and $U_{v}^{\ast} ( x ) >U_{v-1}^{ ( 2 ) } ( x ) $ if $0< x<\sqrt{8 ( v+2 ) ( v+3 ) }$. In fact, some elementary computations give

$$\begin{aligned}& \begin{aligned} U_{v}^{\ast} ( x ) -U_{v-1}^{ ( 2 ) } ( x ) &=v+\sqrt{\frac{2v+5}{2 ( v+2 ) }x^{2}+ ( v+2 ) ^{2}}- \biggl( v-1+\sqrt{\frac{v+3}{v+2}x^{2}+ ( v+3 ) ^{2}} \biggr) \\ &=\sqrt{\frac{2v+5}{2 ( v+2 ) }x^{2}+ ( v+2 ) ^{2}}+1-\sqrt{ \frac{v+3}{v+2}x^{2}+ ( v+3 ) ^{2}}, \end{aligned} \\& \begin{aligned} & \biggl( \sqrt{\frac{2v+5}{2 ( v+2 ) }x^{2}+ ( v+2 ) ^{2}}+1 \biggr) ^{2}- \biggl( \frac{v+3}{v+2}x^{2}+ ( v+3 ) ^{2} \biggr) \\ &\quad=2\sqrt{\frac{2v+5}{2 ( v+2 ) }x^{2}+ ( v+2 ) ^{2}}- \frac{4 ( v+2 ) ^{2}+x^{2}}{2 ( v+2 ) }, \end{aligned} \\& \biggl( 2\sqrt{\frac{2v+5}{2 ( v+2 ) }x^{2}+ ( v+2 ) ^{2}} \biggr) ^{2}- \biggl( \frac{4 ( v+2 ) ^{2}+x^{2}}{2 ( v+2 ) } \biggr) ^{2}=- \frac{1}{4}x^{2}\frac {x^{2}-8 ( v+2 ) ( v+3 ) }{ ( v+2 ) ^{2}}. \end{aligned}$$

It thus can be seen that the upper bound $U_{p}^{\ast} ( x ) $ for $v-1/2< p<\min \{ v+1/2,2v+2 \} $ with $v>-2$ belongs to the new type of bounds $p+r\sqrt{x^{2}+q^{2}}$ ($r>0$) for $W_{v} ( x ) $.

Let us return to Proposition 4.1 again. We note that the number $\lambda_{p}$ is also not computable in the case of

$$ ( p,v ) \in E_{4}\cap \{ p>2v+2,v>-2 \} = \biggl\{ 2v+2< p< v+ \frac{1}{2},-2< v< -\frac{3}{2} \biggr\} . $$

If a better upper estimation $\lambda_{p}^{\ast\ast}>0$ holds for $\lambda_{p}$, then by Proposition 4.1 we can obtain some bounds $W_{v} ( x ) $ similar to the double inequality (4.19), which also implies the new type of bounds. In fact, by the same technique as the proof of Proposition 4.12, we can prove the following proposition, but omit all the details of the proof.

#### Proposition 4.16

*Let* $2v+2< p< v+1/2$ *with* $-2< v<-3/2$. *Then the double inequality*

4.27 $$ p-\sqrt{\lambda_{p}^{\ast\ast}x^{2}+ ( 2v+2-p ) ^{2}}< W_{v} ( x ) < p+\sqrt{\lambda_{p}^{\ast\ast }x^{2}+ ( 2v+2-p ) ^{2}} $$

*holds for* $x>0$, *where*

$$ \lambda_{p}^{\ast\ast}=\min \biggl\{ \frac{v+1/2-p}{v+2}+ \frac {4v^{2}+18v+21}{4 ( v+2 ) ( v+3 ) },\frac {1}{2 ( v+2 ) } \biggr\} . $$

From Proposition 4.10, we know the number $\theta_{p}=1$ for $p\geq c_{v}^{\ast}=v+1/ ( 2v+5 ) $ with $-2< v<-3/2$. It remains to estimate $\theta_{p}$ for $p\in{}[ v+1/2,c_{v}^{\ast})$ with $-2< v<-3/2$. By a similar technique as the proof of Proposition 4.12, we have

4.28 $$ \theta_{p}< \theta_{p}^{\ast}=\min \biggl\{ \frac {4v^{2}+18v+21}{4 ( v+2 ) ( v+3 ) },\frac{c_{v}^{\ast}-p}{v+2}+1 \biggr\} $$

for $p\in{}[ v+1/2,c_{v}^{\ast})$ with $-2< v<-3/2$. Thus, by Proposition 4.1 we conclude the following proposition.

#### Proposition 4.17

*Let* $v+1/2\leq p< c_{v}^{\ast}=v+1/ ( 2v+5 ) $ *with* $-2< v<-3/2$. *Then the double inequality*

4.29 $$ p-\sqrt{\theta_{p}^{\ast}x^{2}+ ( 2v+2-p ) ^{2}}< W_{v} ( x ) < p+\sqrt{\theta_{p}^{\ast}x^{2}+ ( 2v+2-p ) ^{2}} $$

*holds for* $x>0$, *where* $\theta_{p}^{\ast}$ *is given in* (4.28).

#### Remark 4.18

Similarly, the lower bounds given by (4.27) and (4.29) are trivial due to the fact that they are weaker than $2v+2$. However, the upper bounds are new ones which belong to the type of bounds $p+r\sqrt{x^{2}+q^{2}}$ ($r>0$).

## Conclusions

This paper is mainly devoted to proving the monotonicity of

$$ F_{p} ( x ) =\frac{ ( W_{v} ( x ) -p ) ^{2}- ( 2v+2-p ) ^{2}}{x^{2}} $$

on $( 0,\infty ) $ for $v>-2$. As one of applications, from this we arrived at the Simpson–Spector-type inequalities for $W_{v} ( x ) $ (4.1) and other new ones (Proposition 4.1 and Corollary 4.3), which immediately led to some known Simpson–Spector-type inequalities.

As more important applications, we reproved some known results and also found a new type of bounds $p+r\sqrt{x^{2}+q^{2}}$ for $W_{v} ( x ) $.

(i) Proposition 4.4 showed that the lower bound

$$ L_{v} ( x ) =v+\sqrt{x^{2}+ ( v+2 ) ^{2}}\quad \text{with }v>-2 $$

for $W_{v} ( x ) $ is the sharpest, which for $v\geq0$, $v>-1$ and $v\geq-3/2$ are known results (see [6, 9, 10]).

(ii) Proposition 4.4 also indicated that both the upper bounds

$$\begin{aligned}& U_{v+1/2}^{ ( 1 ) } ( x ) = v+\frac{1}{2}+\sqrt {x^{2}+ \biggl( v+\frac{3}{2} \biggr) ^{2}}\quad \text{with }v\geq -\frac{3}{2}, \\& U_{v-1}^{ ( 2 ) } ( x ) = v-1+\sqrt{\frac {v+3}{v+2}x^{2}+ ( v+3 ) ^{2}}\quad\text{with }v>-2 \end{aligned}$$

for $W_{v} ( x ) $ are the sharpest, where the former appeared in [6] and for $v\geq0$ and $v>-1$ was proved in [9, 10], while the latter is a new comer and belongs to the type of bounds $p+r\sqrt{x^{2}+q^{2}}$ ($r>0$).

(iii) We obtained in Proposition 4.10 a new Amos-type bound for $W_{v} ( x ) $, that is,

$$ W_{v} ( x ) < p+\sqrt{x^{2}+ ( 2v+2-p ) ^{2}} $$

holds for $x>0$ and $p\geq c_{v}^{\ast}=v+1/ ( 2v+5 ) $ with $-2< v<-3/2$.

(iv) For $v-1/2< p<\min \{ v+1/2,2v+2 \} $ with $v>-2$, the number $\lambda_{p}$ given in Proposition 4.1 is in general not computable. But by replacing $\lambda_{p}$ with $\lambda_{p}^{\ast}$ defined by (4.21), we gave in Proposition 4.12 a class of elementary function upper bounds

$$ W_{v} ( x ) < U_{p}^{\ast} ( x ) =p+\sqrt{ \frac {4v+5-2p}{2 ( v+2 ) }x^{2}+ ( 2v+2-p ) ^{2}}. $$

As mentioned in Remark 4.15, as an upper bound, $U_{p}^{\ast } ( x ) $ is in general not comparable with other two sharpest upper bounds $U_{v+1/2}^{ ( 1 ) }$ for $v\geq-3/2$ and $U_{v-1}^{ ( 2 ) } ( x ) $ for $v>-2$, and belongs to the new type of bounds $p+r\sqrt{x^{2}+q^{2}}$ ($r>0$).

(v) Using the same technique as Proposition 4.12, we established two new double inequalities for $W_{v} ( x ) $ in the cases of $2v+2< p< v+1/2$ and $v+1/2\leq p< c_{v}^{\ast}$ for $-2< v<-3/2$, that are, (4.27) and (4.29). However, the lower bounds given in (4.27) and (4.29) for $W_{v} ( x ) $ are trivial since they are weaker than $2v+2$. The upper bounds belong to the type of bounds $p+r\sqrt{x^{2}+q^{2}}$ ($r>0$).

Additionally, as a consequence of our results, we deduced some new inequalities for $W_{v} ( x ) $, for example, (4.18), (4.26), and also reobtained some known important inequalities, such as the inequalities proved by Amos [10], Yuan and Kalbfleisch [12, (A.5)], Laforgia and Natalini [14, Theorem 1.1], Segura [15, (61)], [4, Appendix] and so on.
